# Overexpressing *CrePAPS* Polyadenylate Activity Enhances Protein Translation and Accumulation in *Chlamydomonas reinhardtii*

**DOI:** 10.3390/md20050276

**Published:** 2022-04-21

**Authors:** Quan Wang, Jieyi Zhuang, Shuai Ni, Haolin Luo, Kaijie Zheng, Xinyi Li, Chengxiang Lan, Di Zhao, Yongsheng Bai, Bin Jia, Zhangli Hu

**Affiliations:** 1 Guangdong Technology Research Center for Marine Algal Bioengineering, College of Life Sciences and Oceanography, Shenzhen University, Shenzhen 518055, China; wangquan@szu.edu.cn (Q.W.); 1900251002@email.szu.edu.cn (J.Z.); shuai.ni@brbiotech.com (S.N.); luohaolin2021@email.szu.edu.cn (H.L.); zhengkaijie@iga.ac.cn (K.Z.); lixinyi2021@email.szu.edu.cn (X.L.); 1900251008@email.szu.edu.cn (C.L.); dilysxiaodi@szu.edu.cn (D.Z.); baiyongsheng2020@email.szu.edu.cn (Y.B.); 2 Key Laboratory of Optoelectronic Devices and Systems of Ministry of Education and Guangdong Province, College of Optoelectronic Engineering, Shenzhen University, Shenzhen 518060, China; 3 Shenzhen Engineering Laboratory for Marine Algal Biotechnology, Longhua Innovation Institute for Biotechnology, Shenzhen University, Shenzhen 518055, China; 4 Southern Marine Science and Engineering Guangdong Laboratory (Guangzhou), Guangzhou 511458, China

**Keywords:** *Chlamydomonas reinhardtii*, protein production, expression platform, CrePAPS, polyadenylate activity, protein accumulation

## Abstract

The alga *Chlamydomonas reinhardtii* is a potential platform for recombinant protein expression in the future due to various advantages. Dozens of *C. reinhardtii* strains producing genetically engineered recombinant therapeutic protein have been reported. However, owing to extremely low protein expression efficiency, none have been applied for industrial purposes. Improving protein expression efficiency at the molecular level is, therefore, a priority. The 3′-end poly(A) tail of mRNAs is strongly correlated with mRNA transcription and protein translation efficiency. In this study, we identified a canonical *C. reinhardtii* poly(A) polymerase (CrePAPS), verified its polyadenylate activity, generated a series of overexpressing transformants, and performed proteomic analysis. Proteomic results demonstrated that overexpressing CrePAPS promoted ribosomal assembly and enhanced protein accumulation. The accelerated translation was further verified by increased crude and dissolved protein content detected by Kjeldahl and bicinchoninic acid (BCA) assay approaches. The findings provide a novel direction in which to exploit photosynthetic green algae as a recombinant protein expression platform.

## 1. Introduction

Recombinant therapeutic protein production is a key aim of modern biotechnology due to huge market demand [1,2,3]. Recombinant protein expression systems have been established using various platforms including eukaryotic yeast, and mammalian and higher plant systems, but the most widely industrialised approaches involve prokaryotic bacteria [4,5,6,7,8]. These platforms have advantages and limitations in terms of expression efficiency, post-translational processing capacity, production cycle/costs, and application scope. In recent years, microalgae have gained increasing interest as potential bioreactors for recombinant protein production, not only due to their rapid reproduction, short growth period, photoautotrophic capability, and clear genetic background, but also because of their naturally high levels of valuable compounds such as pigments, long-chain polyunsaturated fatty acids, and polysaccharides [9].

The model microalgae *Chlamydomonas reinhardtii* is a preferred recombinant protein production host due to its many beneficial attributes, such as advanced genome informatics for both the nucleus and organelles, stable and convenient nuclear and plastid transformation systems, rapid transformant screening, and potential for both phototrophic or heterotrophic growth [10,11,12,13,14,15]. To date, dozens of recombinant proteins including antibodies, antigens, immunotoxins, enzymes, and therapeutic proteins with commercial value have been successfully expressed in *C. reinhardtii* [16,17,18,19,20]. However, no industrial-scale applications have been established due to inefficient expression and low target protein yield. To increase recombinant protein production in this organism, manipulating transcriptional or translational level regulation may have potential.

The 3′-end polyadenylates of mRNAs in eukaryotic cells appear to share a universal post-transcriptional modification system, and this is believed to impact mRNA expression efficiency and protein translation abundance [21]. The poly(A) tail serves three major functions: stabilising ribonucleic acids in the cytoplasm, facilitating the export of mRNAs from the nucleus, and elevating gene expression efficiency. A recent study revealed that poly(A) tails and attached poly(A)-binding proteins (PABPs) may also be involved in regulating translation termination [22]. In the cytoplasm of eukaryotic cells, long-tail mRNAs, such as mRNAs with 70–80 nucleotide tails in yeast and mRNAs with ~250 nucleotides tails in mammalian cells, appear to last longer than their shorter peers, presumably because mRNAs are usually shortened from their 3′-ends gradually [23]. Additionally, the degradation mechanism of mRNAs does not occur unless they have been shortened to a specific length. For instance, in mammals, when the poly(A) tails of mRNAs are shortened to less than 27 adenylates, dissociation of PABP occurs, followed by recruitment of the terminal uridylyl transferases TUT4 and TUT7 that mediate mRNA 3′ uridylation degradation [24,25,26]. The length of poly(A) tails determines the stability and half-life of mRNAs; slower degradation and a longer half-life increase the opportunity of being recognised by the ribosome machinery, which could enhance the accumulation of expressed proteins.

Furthermore, once a poly(A) tail is formed in the cytoblast, nuclear PABPs recognise and attach to it. These PABPs are often referred to as PABPNs in order to distinguish them from cytoplasmic poly(A)-binding proteins (PABPC) [22,27,28]. PABPNs are often known as pivotal factors mediating mRNA transportation from the nucleus to the cytoplasm [29]. Replacing PABPNs with PABPCs can occur after mRNAs are transported into the cytoplasm, especially during the first round of mRNA translation, which appears to promote the process. Thus, PABPN and PABPC conversion signals might be related to the translation efficiency of a transcript [30,31]. 

The length of poly(A) tails and their attached PABPs may also boost expression efficiency. Although the specific mechanism needs to be further investigated, polyadenylated RNAs are reported to undergo more efficient translation than deadenylated RNAs [32,33]. Moreover, RNAs with longer poly(A) tails are usually translated more effectively than those with shorter tails [34,35]. A possible explanation is that interaction between PABP and eIF4G, a translation initiation factor binding to the 5′ cap, probably directly affects the protein-coding efficiency [36]. Based on previous research, a stable and suitable length of poly(A) tail and its combination with PABPs can indeed augment mRNA expression efficiency, at least at the molecular level, and a similar mechanism could be exploited to facilitate future recombinant protein production in the industry. 

Canonical polyadenylate polymerases (*cPAPSs*) are genes that encode eukaryotic poly(A) polymerases directing the synthesis of poly(A) tails at the 3′-end of all transcripts. There is a close relationship between the PAPS gene and the polyadenylate tail that is universally recognised, but the underlying molecular mechanisms need to be explored before they can be exploited. Decades have passed since the first *cPAPS* gene (Pap1) was cloned in *Saccharomyces cerevisiae*, and various *cPAPS* genes have since been identified and functionally characterised in many species [37,38,39,40,41]. Although the *cPAPS* copy number is not strictly positively correlated with the development of an organism, the vast majority of known lower eukaryotic cells, including yeast and most algae, have only one *cPAPS*, in contrast to the multiple functionally specialised poly(A) polymerases in higher eukaryotes [38,39]. For instance, three PAPSs have been found in humans (PAPα, PAPβ, and PAPγ). PAPα contains a C-terminal regulatory region next to the highly conserved catalytic N-terminal domain that functions in both the nucleus and cytoplasm, whereas PAPγ is only located in the nucleus. In contrast to PAPα and PAPγ, which are present in somatic cells, PAPβ lacks the C-terminal region, is exclusively cytoplasmic, and is only found in testis cells [42]. 

Functionally specialised *PAPSs* are also present in plants, and they are linked to antagonistic control of flowering time [43]. There are four *cPAPS* genes (*PAPS1* to *PAPS4*) reported in *Arabidopsis*. The protein product of *PAPS3* is similar to PAPβ, it lacks the extended C-terminal region, it is located in the cytoplasm, and it is mainly expressed in pollen. By contrast, PAPS1, PAPS2, and PAPS4 contain the extended C-terminal region, are exclusively located in the nucleus and are expressed throughout the whole plant [39,44]. It was proposed that the cooperation of multiple PAPS isomers provides an additional layer of expression regulation in plants [43,45,46]. How this additional expression regulation mechanism can be exploited for recombinant protein production deserves attention. 

The single-celled model alga *C. reinhardtii* has only one copy of *cPAPS* in the genome, making it ideal for investigation. Furthermore, *C. reinhardtii* is considered a promising platform for recombinant protein production. Thus, in this study, we explored the intrinsic relationship between excessive polyadenylate activity and protein translation and accumulation in *C. reinhardtii* by overexpressing the *CrePAPS* gene. Transformants overexpressing *cPAPS* were subjected to proteomic analysis to investigate whether increasing polyadenylate activity could increase protein production. Additionally, we explored the potential molecular mechanism by which PAPS regulates protein expression, and evaluated the possibility of increasing the recombinant protein yield using a microalgae platform.

## 2. Results

### 2.1. Generation of CrePAPS-Overexpressing Lines and Identification of Insertion Mutants

**Figure 1:**
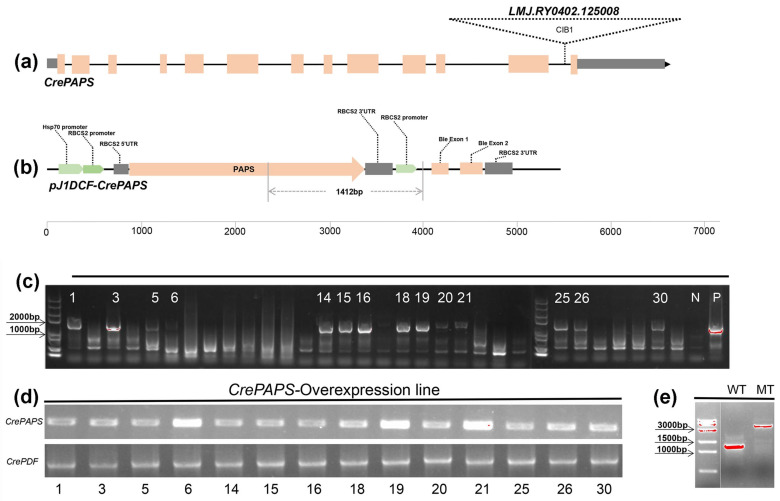
Generation and confirmation of *CrePAPS*-overexpressing *Chlamydomonas* *reinhardtii* transgenic lines. (**a**) The *CrePAPS* gene structure and the *CIB1* cassette insertion position. (**b**) Schematic diagram of the *pJ1DCF-CrePAPS* vector. Promoters and other components are labelled. Primer locations and the size of the corresponding amplicon used to screen transformants are indicated by grey lines. (**c**) Agarose gel electrophoresis showing whether the *HSP70A-RBCS2:CrePAPS* cassette is integrated with the *C. reinhardtii* genome. WT and *pJ1DCF-PAPS* plasmids were included as negative (N) and positive (P) controls, respectively. Strains marked with numbers are positive transformants. (**d**) Semi-quantitative detection of *CrePAPS* expression levels in 14 positive transformants. (**e**) Amplicon of the *CIB1* cassette-inserted mutant and the corresponding amplicon in the WT strain.

Canonical *C. reinhardtii* poly(A) polymerase (*CrePAPS*) genes were screened based on NTP_transf_2, PAP_RNA-bind and PAP_central (PF01909, PF04926 and PF04928) conserved motifs. The search identified a 6894 base pair (bp) gene locus on chromosome 10 (Cre10.g433750) that was predicted to be our target gene. The 2412 bp coding sequence of *CrePAPS* is divided into 13 exons (Figure 1a). Since there is only one copy of this gene in the genome, altering the expression pattern or enzyme activity of *CrePAPS* may have a relatively large impact on normal growth and development, as well as on the expression profile.

To evaluate the effects of *CrePAPS* on transcription in algae, we constructed the *pJ1DCF-CrePAPS* plasmid (Figure 1b) and transformed it into the wild-type (WT) strain to generate the *CrePAPS*-overexpressing (OE) line. The coding sequence of *CrePAPS* is under the control of the chimeric constitutive tandem *HSP70A-RBCS2* promoter and *RBCS2* 3‘-UTR is used as a termination signal for *CrePAPS* transcription. An independent *RBCS2* promoter drives the bleomycin resistance gene *ble* as a selection marker. The resulting transformants were spread on a plate with antibiotics to obtain candidate colonies. Correct insertion of the *HSP70-RBCS2:CrePAPS* cassette in the nuclear genome of candidate colonies was confirmed by genomic PCR analysis with gene-specific primers (Figure 1b and Table S1). A total of 14 transformants were identified by the presence of bands of the expected size (1412 bp) as observed in positive controls (the *HSP70-RBCS2:CrePAPS construct*) and the absence of bands in WT controls (Figure 1c). A semi-quantitative PCR was used to confirm and quantify the level of transcription of *CrePAPS* in all 14 correctly inserted strains. OE6, 19, and 21 exhibiting high *CrePAPS* expression levels were selected for further studies (Figure 1d). Additionally, a 2223 bp *CIB1* cassette-inserted *CrePAPS* mutant (*LMJ.RY0402.125008*) strain (MT) was also identified and used in this study (Figure 1e).

**Figure 2:**
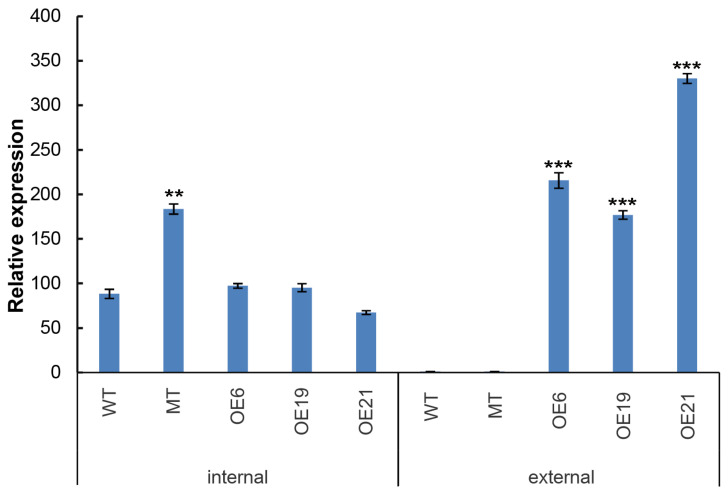
Quantitative analysis of internal and external expression of *CrePAPS* in WT, MT, OE6, OE19, and OE21. Bars represent average values of three replicates ± standard deviation (SD). All expression levels were normalised against expression levels of *CrePP2A* (protein phosphatase 2A). *** and ** indicate significant differences compared to internal expression in WT.

To further determine *CrePAPS* transcript levels, quantitative PCR (qPCR) was used to detect both internal and external expression of *CrePAPS* in OE6, OE19, OE21, MT, and WT lines (Figure 2). The results showed no significant differences in the internal expression of *CrePAPS* among OE6, OE19, OE21, and WT. However, the internal expression of *CrePAPS* in MT was almost twice that in WT. The enhancement is probably due to the strong promoter of the *CIB1* cassette. This indicates that MT is also an overexpression strain. To investigate the external expression of *CrePAPS*, reverse primers corresponding to the *RBCS2 3′-UTR* region were employed. As expected, external expression was not found in WT or MT. The external expression levels of *CrePAPS* in all three transformants were more than twice that of *CrePAPS* in WT. Eventually, we obtained four overexpressing lines, including one internal and three external strains.

### 2.2. Analysis of CrePAPS Polyadenylate Activity

**Figure 3:**
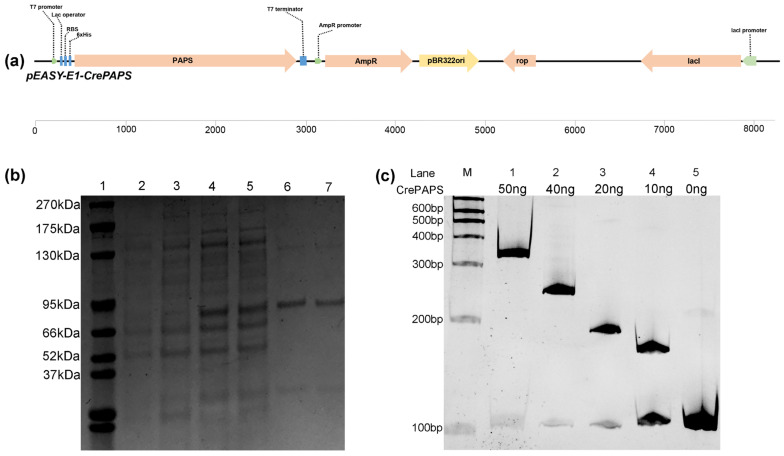
In vitro analysis of *CrePAPS* polyadenylate activity. (**a**) Schematic diagram of the vector used to generate CrePAPS protein. (**b**) SDS-PAGE image showing prokaryotic expression of CrePAPS. Lane 1, protein ladder; lane 2 and 3, total protein extracted from non-induced *E. coli BL21*/*pEASY-E1-CrePAPS*; lane 4 and 5, total protein extracted from IPTG-induced *E. coli BL21/pEASY-E1-CrePAPS*; lane 6 and 7, purified CrePAPS. (**c**) Autoradiograph of the in vitro polyadenylation assay. Lane 5 corresponds to the unpolyadenylated RNA substrate. M indicates a RNA ladder. Lane 1–4, 50 ng, 40 ng, 20 ng, and 10 ng of purified CrePAPS incubated with pre-mRNA; lane 5, ultra-pure water incubated with pre-mRNA (negative control).

In order to verify the polyadenylate activity of CrePAPS, the *pEASY-E1-CrePAPS* plasmid containing the 2412 bp full-length CrePAPS cDNA under the control of the T7 promoter was constructed (Figure 3a) and transformed into the *Escherichia coli* BL21 strain. Transformants expressed CrePAPS protein with a molecular weight of 84.9 kDa, which was purified by Ni-NTA resin (Figure 3b). Purified protein was used to perform in vitro polyadenylation assays. A pre-mRNA of a specific length was transcribed and used to assess the polyadenylate activity of CrePAPS. Additionally, to assess whether the length of the poly(A) tail is associated with the amount of protein, a serial dilution of known amounts of purified protein (50 ng, 40 ng, 20 ng, and 10 ng) was prepared. The results showed that CrePAPS indeed exhibited polyadenylate activity because the mRNA length in vitro could be extended by adding CrePAPS protein (Figure 3c). Additionally, a ~200 bp longer poly(A) tail could be obtained by increasing the amount of protein from 0ng to 50ng. Thus, we anticipated similar results when overexpressing the *CrePAPS* gene in the unicellular green alga *C.* *reinhardtii*.

### 2.3. Label-Free Quantitative Proteomic Analysis of C. reinhardtii Strains Exhibiting Higher Polyadenylate Activity

Triplicate protein samples of OE6, OE19, OE21, MT, and WT were subjected to LC-MS/MS analysis. Data are expressed as average label-free quantification (LFQ) values from parallel preparations for each strain. Unique peptide sequences were analysed quantitatively and qualitatively using a Thermo Fisher Orbitrap Fusion Eclipse mass spectrometer. Protein identification was performed using the UniProt database. Available online: http://www.uniprot.org (accessed on 6 March 2022). R^2^ > 0.99 for ion intensity among three biological samples for each strain in linear regression analysis of peptide distribution indicated that the experiments had good overall reproducibility. Peptide identification and intensity information from all samples were assembled into a single peptide array after PEAKS X searching against the UniProt database, whereupon 3613 proteins with a unique peptide score ≥1 were functionally assigned. Unique identified proteins for each sample are listed in Table 1. In summary, three parallel samples identified 2262, 2187, and 2188 proteins in MT; 2452, 2376, and 2399 proteins in WT; 2197, 2181, and 2203 proteins in OE6; 2178, 2168, and 2199 proteins in OE19; and 2494, 2492 and 2553 proteins in OE21. Quantitative values for each identified protein were also obtained based on ion intensity changes (Table S2).

### 2.4. Screening Differentially Accumulated Proteins (DAPs) and Proportionally Altered Proteins (PAPs)

**Figure 4:**
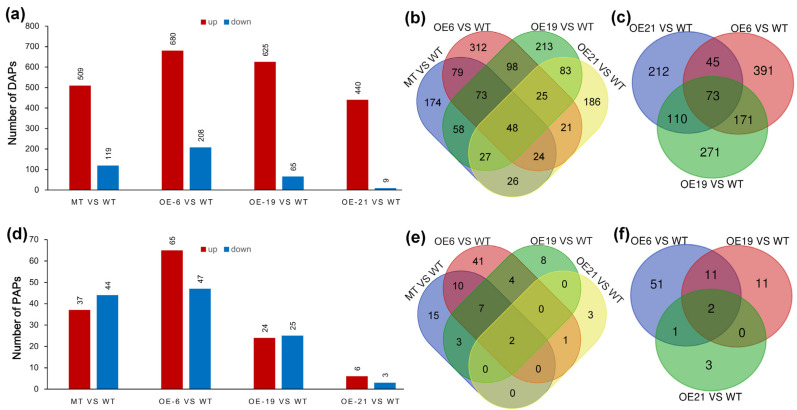
Screening of differentially accumulated proteins (DAPs) and proportionally altered proteins (PAPs). (**a**) Total DAPs screened in all strains. (**b**) Venn diagrams of up-DAPs for MT vs. WT, OE6 vs. WT, OE19 vs. WT, and OE21 vs. WT. (**c**) Venn diagrams of up-DAPs for OE6 vs. WT, OE19 vs. WT, and OE21 vs. WT (**d**) Total PAPs screened in all strains. (**e**) Venn diagrams of up-PAPs for MT vs. WT, OE6 vs. WT, OE19 vs. WT, and OE21 vs. WT. (**f**) Venn diagrams of up-PAPs for OE6 vs. WT, OE19 vs. WT, and OE21 vs. WT.

To explore whether superfluous CrePAPS activity could induce abnormal protein accumulation, the amount of each unique protein in four OE lines was compared with that in WT cells. DAPs were identified based on >2-fold differences compared to WT and *p* ≤0.05 (*t*-test) according to pre-filtered quantitative data. Protein candidates detected in at least two of three replicates were retained for further analysis, while those detected in none or only one of three replicates were removed. The results revealed 628 (509 increased and 119 decreased), 888 (680 increased and 208 decreased), 690 (625 increased and 65 decreased), and 449 (440 increased and 9 decreased) DAPs in MT vs. WT, OE6 vs. WT, OE19 vs. WT and OE21 vs. WT, respectively (Figure 4a, Table S3). The vast majority of DAPs were upregulated, as expected. We speculated that excessive polyadenylation at the cellular level would increase the stability of mRNAs, prolong their half-lives, and further increase their chances of being recognised and translated by ribosomes. Therefore, a Venn diagram analysis was performed on these upregulated DAPs (up-DAPs; Figure 4b,c). The results showed that there were 1447 up-DAPs in total, of which 48 were synchronously upregulated in all four OE lines. Even excluding the MT strain, there were still 1273 up-DAPs, 73 of which were upregulated among three external OE lines.

Since poly(A) polymerase is an enzyme that universally prolongs poly(A) tail length, it is likely to increase the expression efficiency of multiple proteins simultaneously. However, in the industrial production of recombinant proteins, expressing and isolating one or a few proteins is typically the focus. Increasing the ratio of target protein vs. total protein makes sense to reduce cost and facilitate separation. Thus, measuring PAPs is useful, especially where proportions increase. Total protein was defined as the sum of 1271 proteins detected simultaneously in WT, MT, OE6, OE19, and OE21 (Table S4). The ratio of each protein in each sample was compared with control groups, and proteins with more than twice the variation in the ratio were screened. The results revealed 82 (37 increased and 44 decreased), 49 (24 increased and 25 decreased), 112 (65 increased and 47 decreased), and 9 (6 increased and 3 decreased) PAPs for MT vs. WT, OE6 vs. WT, OE19 vs. WT and OE21 vs. WT, respectively (Figure 4d and Table S5). Similarly, Venn diagram analysis was performed on up-PAPs, and the results showed that 94 PAPs had a ratio increase, including two PAPs whose content increased concurrently among four OE strains. Excluding insertion mutants, two up-PAPs remained. 

In summary, although these four OE lines expressed *CrePAPS* at high levels, the identified DAPs and PAPs were divergent. Simultaneous up-DAPs and up-PAPs represent only a small fraction of total DAPs and PAPs. This suggests that there may be some other mechanism involved in *PAP*-mediated protein accumulation, which leads to inconsistent PAPs and DAPs.

### 2.5. Gene Ontology (GO) and Kyoto Encyclopedia of Genes and Genomes (KEGG) Functional Annotation Analyses of Upregulated DAPs and PAPs

**Figure 5:**
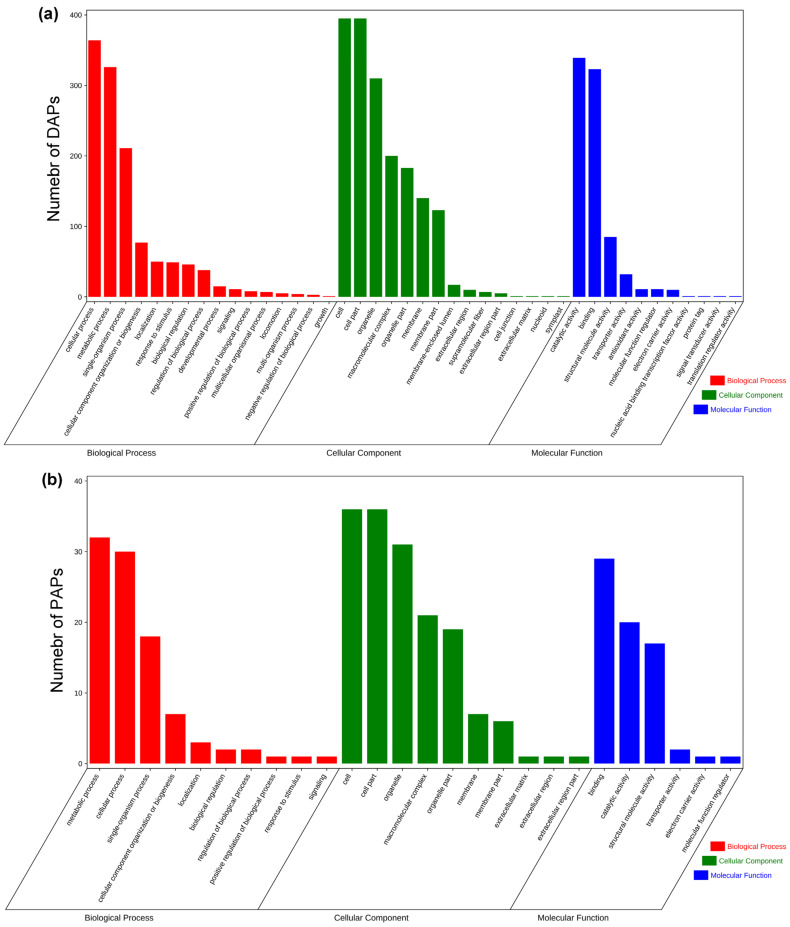
Gene ontology (GO) profiles of upregulated DAPs and upregulated PAPs. (**a**) GO profiles of DAPs. (**b**) GO profiles of PAPs.

To further investigate the effect of excess polyadenylate activity and facilitate screening of possible industrialised recombinant proteins, GO analysis was performed on 1447 and 94 upregulated DAPs and PAPs, respectively, based on biological processes, cellular components, and molecular functions. In total, 86 biological processes, 88 cellular components, and 17 molecular function clusters displayed obvious enrichment with *q* < 0.05 according to the false discovery rate (FDR) for up-DAPs (Table S6). Meanwhile, 27 biological processes, 29 cellular components, and 8 molecular function clusters were obviously enriched for up-PAPs (Table S7). The most enriched categories of up-DAPs and up-PAPs are listed according to the number of proteins. As shown in Figure 5a,b, the top three enriched clusters are exactly the same for up-DAPs and up-PAPs, including cellular processes (GO:0009987), metabolic process (GO:0008152), and single-organism process (GO:0044699) in the biological processes category; cell (GO:0005623), cell part (GO:0044464) and organelle (GO:0043226) in the cellular component category; and binding (GO:0005488), catalytic activity (GO:0003824) and structural molecule activity (GO:0005198) in the molecular function category. Moreover, some proteins related to intracellular protein hyperaccumulation, such as ribosome assembly, ATP synthesis, and protein translation, were enriched for up-DAPs and up-PAPs. These clusters were ribosome (GO:0005840), ribosomal subunit (GO:0044391), large ribosomal subunit (GO:0015934), small ribosomal subunit (GO:0015935), oxidoreductase activity (GO:0016491), organonitrogen compound biosynthetic process (GO:1901566) and translation (GO:0006412). These enriched results implied that when CrePAPS is overexpressed in organisms, ribosomal proteins are highly translated to increase the overall translation level. Additionally, to adapt to the consumption of the translation machinery, some proteins related to oxidative phosphorylation are also strongly upregulated.

**Figure 6:**
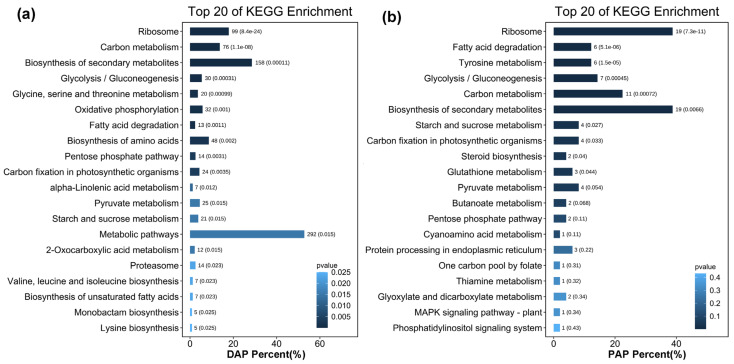
Enriched KEGG pathways of up-DAPs and up-PAPs. (**a**) Enriched KEGG pathways of up-DAPs. (**b**) Enriched KEGG pathways of up-PAPs.

Proteins in the same pathway often perform their biological functions in a concerted manner. Pathway enrichment analysis using the KEGG database was performed to characterise the potential biological functions of proteins that were induced by overexpression of CrePAPS (Figure 6). Upregulated proteins, both up-DAPs and up-PAPs, in the most enriched pathways were clustered into a ribosome (ko03010), implying increased overall translation. Additionally, upregulation of proteins involved in fatty acid degradation (ko00071) and carbon metabolism (ko01200) pathways indicated that the accumulation of proteins may be accompanied by increased fatty acid and carbon source consumption. Increased expression of multiple proteins involved in the biosynthesis of secondary metabolites (ko01110) was also noted, which might reflect the increased demand for protein processing alongside elevated protein translation.

### 2.6. Subcellular Localisation of Up-DAPs and Up-PAPs

**Figure 7:**
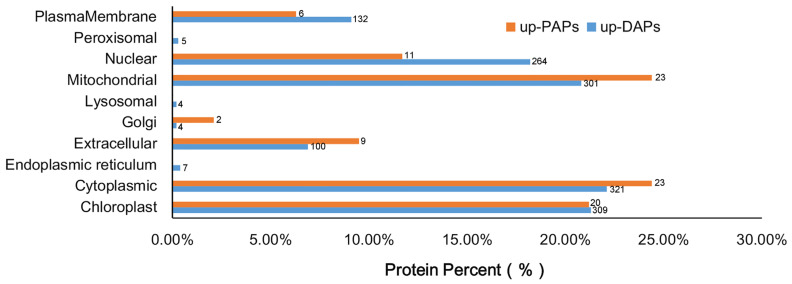
Subcellular localisation of up-DAPs and up-PAPs according to CELLO prediction.

Overexpression of CrePAPS may lead to increased overall protein translation, which may prove useful for future industrialisation of recombinant proteins. However, in order to rapidly identify target proteins and establish appropriate purification systems, prediction of subcellular localisation for up-DAPs and up-PAPs is necessary. Hence, the amino acid sequences of 1447 up-DAPs and 94 up-PAPs were downloaded and online subcellular localisation prediction was performed by CELLO Available online: http://cello.life.nctu.edu.tw/ (accessed on 5 March 2022). The results showed that up-DAPs were widely distributed almost everywhere in the cell, including the nucleus (264), cytoplasm (321), endocellular organelles such as chloroplasts (309), mitochondria (301), protein processing regions including the Golgi complex (4) and endoplasmic reticulum (7), outside cells in the plasma membrane (132) and extracellular space (100), and in microbodies such as peroxisomes (5) and lysosomes (4). Interestingly, up-PAPs were not as widely distributed as up-DAPs, and were found in only seven positions, namely chloroplasts (20), cytoplasm (23), extracellular (9), Golgi (2), mitochondria (23), nucleus (11) and plasma membrane (6) (Figure 7).

Although there were slight differences, up-DAPs and up-PAPs were located almost everywhere in the cell following alteration of cellular polyadenylate activity. In summary, cytoplasmic, chloroplast and mitochondrial proteins accounted for 22.1%, 21.3%, and 20.8% of up-DAPs and 24.4%, 21.2%, and 24.4% of up-PAPs, respectively, which might provide a reference for selecting protein targets for industrialisation.

### 2.7. Analysis of Crude total Protein Content and Dissolved Protein Content

**Figure 8:**
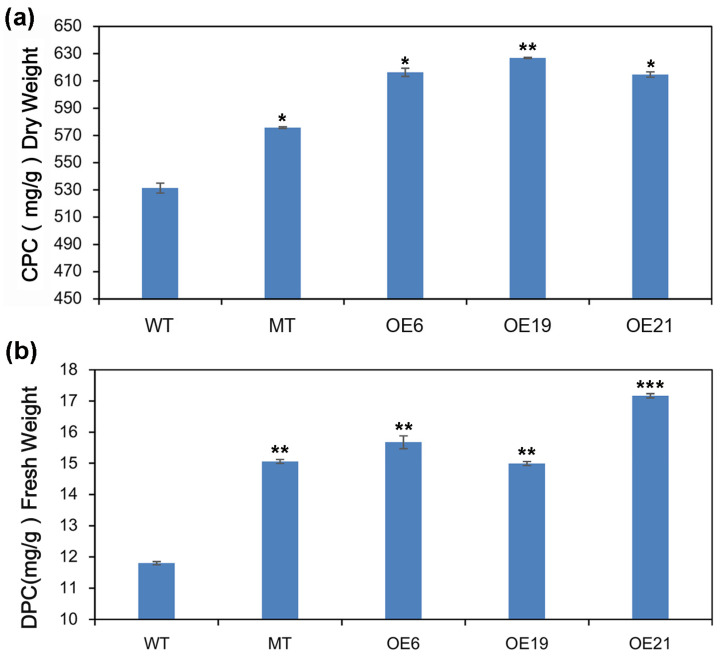
Analysis of protein accumulation. (**a**) Crude protein content of WT, MT, OE6, OE19, and OE21 algae cells measured by the Kjeldahl method. (**b**) Dissolved protein content of WT, MT, OE6, OE19, and OE21 algae cells measured by the BCA method. Data are expressed as means ± standard error (SE; *n* = 3). Asterisks represent significant differences compared with WT (*t*-test: * *p* < 0.01; ** *p* < 0.05; *** *p* < 0.001).

In order to further determine the changes in protein content in green algae cells following overexpression of *CrePAPS*, crude and dissolved protein content were measured using the Kjeldahl and BCA methods. Final crude protein content was estimated using a general conversion factor of 6.25. In this way, the crude protein content of WT, MT, OE6, OE19, and OE21 was estimated as 531.36, 575.73, 616.28, 626.85, and 614.67 mg/g dry weight (Figure 8a). 

The BCA method yielded dissolved protein content values for WT, MT, OE6, OE19, and OE21 of 11.80, 15.06, 15.67, 14.99, and 17.17 mg/g fresh weight (Figure 8b). It was found that both crude and dissolved protein contents were increased in all four OE strains. This result further confirms that excessive CrePAPS activity promotes protein translation and accumulation in *C. reinhardtii*.

## 3. Discussion

Microalgae such as the unicellular green alga *C. reinhardtii* have many advantages that make them ideal for recombinant protein production [47,48]. However, low yield and inefficient downstream processing are the main obstacles to engineering algae strains for industrialisation [49,50]. Many approaches for increasing recombinant protein yield have been reported, including improved process techniques, specific environmental stress treatments, and protein production cell line optimisation [51,52,53]. Improving protein expression efficiency via translation regulation could also help to overcome these obstacles. For example, a recent study showed that directed evolution of rRNA can improve translation kinetics and recombinant protein yield [54]. Polyadenylating the 3′ end of mRNA can stabilise and enhance translation. However, little consideration has been given to the potential industrial application of polyadenylate modification on translation regulation. Therefore, we conducted an explorative study focusing on the principal constituents of the intracellular polyadenylate machinery, namely poly(A) polymerase, and how it affects protein accumulation at the proteome level when overexpressed. 

In the present study, the canonical poly(A) polymerase gene was identified in *C.* *reinhardtii*, and its polyadenylate activity was verified. In vitro polyadenylation assays proved that increasing the abundance of CrePAPS protein over a certain range could prolong the poly(A) tail length, resulting in a longer mRNA. An extended poly(A) tail enhances the binding of PABs, slows degradation, and increases the chance of being recognised by the translation machinery [55,56]. Modifying the 3′ end of mRNA is essentially a transcriptional regulatory mechanism [25]. Similar prolongation of the poly(A) tail and improvement of translation efficiency are likely to be of value in vivo. Hence, an extra copy of the *CrePAPS* gene was integrated into the *C.* *reinhardtii* genome, and its expression was driven by the powerful chimeric promoter *HSP70-RBCS2*. Finally, three external *CrePAPS*-overexpressing transformants and an internal *CrePAP*S overexpression mutant were obtained. qRT-PCR results verified that the expression levels of *CrePAPS* in these four lines exceeded that of the WT strain. SDS-PAGE and silver staining were performed to detect whether the amount of CrePAPS protein was increased in OE strains (Figure S1). However, no clear bands of the expected size for CrePAPS were observed. We speculated that the reason might be the low background level of CrePAPS protein, even after overexpression, the expression level exceeded the resolution of SDS-PAGE detection. In general, increased transcription generates more protein products, and this should result in higher polyadenylate activity in the cell [57], and excess polyadenylate activity is reported to increase protein accumulation [58,59,60]. 

LC-MS/MS analysis was carried out on these *CrePAPS* OE strains to characterise the proteome profile and verify the promoting effect on protein translation. Proteomic results demonstrated that levels of numerous proteins were obviously increased following overexpression of *CrePAPS*. In particular, the upregulation of DAPs (Section 2.4) surprised us. Many proteins (>440) were increased by two-fold or more compared to WT in the four tested strains, accounting for about a quarter of the total identified proteins, whereas very few DAPs were downregulated. This near-universal upregulation suggests that polyadenylate modification is a powerful transcriptional regulation process that may bypass some conventional gene regulation mechanisms. However, some downregulated DAPs were identified, indicating that this process itself may also be regulated by other mechanisms. Regardless, there were many more up-DAPs than down-DAPs, indicating that this type of modification is likely to be useful for protein production industrialisation. 

Increasing the ratio of target protein vs. total protein–protein is conducive to industrial production and purification. Although this ratio was enhanced for relatively few proteins, due to the universal increase in the accumulation of many proteins, many PAPs were upregulated (Table S5). In addition, it was noticed that less than a fifth of DAPs were upregulated in OE lines, and many up-DAPs were specific to certain OE lines. Although CrePAPS activity was enhanced, it affected the translation of four OE lines in a different manner. These differences may be caused by diverse expression patterns among different lines, or some unknown regulatory mechanisms [61,62].

The results from GO and KEGG enrichment analysis of up-DAPs and up-PAPs strengthen the evidence that the total translational levels in *CrePAPS* OE lines were improved. The most obvious enrichment categories and pathways were associated with ribosome assembly, based on both GO and KEGG analyses. Upregulation of the ribosomal machinery seems to be conducive to protein translation and accumulation. Simultaneously, crude protein and dissolved protein assays further proved that excessive CrePAPS activity could result in more protein accumulation in *C.* *reinhardtii*. Subcellular location analysis of up-DAPs and up-PAPs indicated that it is better to choose cytoplasmic, chloroplast, and mitochondrial proteins for recombinant protein expression in *CrePAPS* OE engineered *C.* *reinhardtii* lines. 

Although the results above are based on endogenous proteins rather than exogenously introduced recombinant proteins, they are still encouraging due to the huge advantages of using unicellular green algae as a platform for recombinant protein production. Our findings will facilitate future use of this high-potential platform.

## 4. Materials and Methods

### 4.1. Strains and Growth Conditions

The wild-type *C. reinhardtii* cc-5325 strain (WT) used in this study was obtained from Guangdong Technology Research Center for Marine Algal Bioengineering. The mutant strain *LMJ.RY0402.125008* (MT) was obtained from the Chlamydomonas Resource Center, University of Minnesota, Minneapolis USA. For data collection, algal cells were grown using Trisacetate-phosphate liquid medium (TAP) in an illumination incubator at 22 °C with shaking at 150 rpm under constant light (40 μmol photons m^−2^ s^−1^). All experiments were performed with cells in the exponential growth phase. Cells were harvested by centrifugation at 3000 rpm for 5 min and washed twice with distilled water

### 4.2. RNA Isolation and qRT-PCR

Total RNA was extracted from cells at the exponential growth phase using an Easy-Pure Plant RNA kit (Transgen, ER301, Beijing, China). RNA quality was assessed using a NanoDrop2000 Ultra Microscope Photometer (Thermo, Massachusetts, USA). First-strand cDNA was prepared using EasyScript One-Step gDNA Removal and cDNA Synthesis SuperMix (Transgen, AE311, Beijing, China) strictly following the manufacturer’s instructions. Gene expression patterns were analysed by qRT-PCR with SYBR Green Master (Roche, 4913914001, Basel, Switzerland) using an ABI QuantStudio 6 Flex Detection Device (Thermo, Massachusetts, USA) as recommended by the manual. Three biological replicates and three technical replicates were included. The *CrePP2A* gene (*PP2A, Cre11.g477300*) served as an internal control, and relative expression levels of target genes were calculated via the 2^−ΔΔCt^ method. Primers used for qRT-PCR analysis are listed in Supplementary Table S1.

### 4.3. Overexpression Plasmid Construction and Transformation

The CrePAPS nucleotide sequence was obtained from Phytozome13. Available online: https://phytozome-next.jgi.doe.gov/info/Creinhardtii_v5_6 (accessed on 10 February 2022) and amplified by reverse transcription PCR from WT *C. reinhardtii* cc-5325 using gene-specific primers (Supplementary Table S1). The coding sequence of CrePAPS was cloned into *Nde*I and *Kpn*I restriction sites of the expression vector *pJ1DC*F harbouring a bleomycin resistance gene cassette driven by the RBCS2 promoter for transformant selection. The constructed *pJ1DCF-CrePAPS* plasmid was transformed into *C. reinhardtii* WT strains via the electroporation method. Algal cells in the mid-exponential phase (approximately 1 − 3 × 10^6^ cells mL^−1^) were collected by centrifugation at 2000 rpm for 10 min and resuspended in TAP medium containing 40 mM sucrose to a final concentration of 108 cells mL^−1^. Cells were incubated at 40 °C with shaking at 350 rpm for 30 min in a shaking incubator. Finally, cells were diluted to 2 × 10^4^ cells mL^−1^ with TAP medium containing 40 mM sucrose and aliquoted into 250 μL per tube. Two micrograms of linearised plasmid DNA was added to each tube and electroporation was performed using a Gene Pulser Cuvette (Bio-Rad, Hercules, CA, USA) at 500 V, 25 μF, and 400 Ω of resistance. After electroporation, cells were transferred to TAP liquid medium containing 40 mM sucrose and recovered for 24 h in a shaker at 120 rpm under continuous white light. The recovered cells were spread on a TAP plate containing 10 μg mL^−1^ zeocin.

### 4.4. Expression and Purification of CrePAPS Proteins in E. coli

The CrePAPS coding region was amplified from the WT cDNA and subcloned into the *pEASY-Blunt E1* Expression Vector (Transgen, CE111, Beijing, China). A 6xHis-tag was fused at the N-terminus of CrePAPS, and gene expression was under the control of the T7 promoter. The constructed plasmid was transformed into *E. coli* BL21 (DE3). Positive transformants were cultured overnight in LB medium with 100 μg/mL ampicillin at 37 °C and 200 rpm. Next, 1 mL of overnight cells was added to 50 mL of LB containing ampicillin and grown to exponential phase (OD600 = 0.4 − 0.6). Subsequently, Isopropyl-beta-D-thiogalactopyranoside (IPTG) was added to the culture to a final concentration of 1.0 mM and the culture was incubated for 4 h at 28 °C and 200 rpm. Cells were harvested by centrifugation at 12,000× *g* and 4 °C for 1 min, and resuspended in 320 μL of phosphate-buffered saline (PBS) buffer (pH 8.0). After treatment with an ultrasonic crushing device and centrifugation at 12,000 rpm and 4 °C for 5 min, the supernatant was collected. Ni-NTA Resin (Transgen, DP101, Beijing, China) was used for purifying the target protein from the collected supernatant following the manufacturer’s instructions.

### 4.5. In Vitro Polyadenylation Assay

An in vitro transcribed pre-mRNA was obtained using a T7 High-efficiency Transcription Kit (Transgen, JT101, Beijing, China) following the manufacturer’s instructions and used to detect polyadenylate activity of *C. reinhardtii* PAPS. Briefly, in vitro reaction mixtures comprised 2.5% (*wt/vol*) polyvinyl alcohol, 8% (*wt/vol*) glycerol, 8 mM HEPES (pH 7.9), 17 mM (NH4)_2_SO_4_, 2 mM MgCl_2_, 0.08 mM EDTA, 0.02 mM DTT, 4 mM creatine phosphate, 50 μM ATP, and 0.5 U RNasin. Reaction mixtures were incubated at 25 °C for 3 h after adding 0.1 pmol precursor RNA and a certain amount of protein. Stop solution consisting of a 220 μL mixture of 20 mM Tris (pH 7.9), 0.1 M NaCl, 10 mM ethylene diamine tetraacetic acid (EDTA), 1% (wt/vol) sodium dodecyl sulfate (SDS), and 40 μg proteinase K was used to halt the polyadenylation reaction. The polyadenylated mRNA was extracted by adding an equal volume of phenol/chloroform, and precipitated by adding two volumes of ethanol containing 1/10 (*vol/vol*) 3 M NaAc. A 6% (*wt/vol*) urea-acrylamide gel was used to measure the extracted RNA length after PAPS processing.

### 4.6. Protein Extraction from Algal Cells

Algal cells at the exponential growth phase were harvested by centrifugation at 3000 rpm for 5 min, washed twice with distilled water, and immediately submerged in liquid nitrogen. Proteins from algal cells were released by sonication on ice in lysis buffer (8 M urea, 1% SDS, 40 mM Tris-base pH 8.5, 10 mM dithiothreitol (DTT), and 2 mM EDTA). Extracted proteins were reduced with 10 mM DTT at 56 °C for 30 min, then alkylated using 20 mM iodoacetamide in a darkroom for 30 min. The reduced and alkylated protein mixtures were precipitated by adding methanol and chloroform. After centrifugation at 12,000× *g* at 4 °C, the pellet was collected, dissolved in ultrapure water, and sonicated on ice. After centrifugation again at 12,000× *g* and 4 °C, an aliquot of supernatant was taken to determine the total protein content using a BCA Protein Quantitation Kit (Transgen, DQ111-01, Beijing, China). 

### 4.7. Protein Digestion and LC- MS/MS Analysis

An aliquot of total protein (20 µg) was removed from each sample and digested with trypsin (Promega, Fitchburg, WI, USA) at 37 °C for 16 h (protein:trypsin = 100:1). The digested solution was passed through a C18 desalting column and subsequently lyophilised to dryness. Digested fractions were resuspended in buffer A (0.1% formic acid) and peptides were collected by centrifugation at 20,000× *g* for 10 min at an estimated concentration of ~0.5 µg/µL. An Orbitrap Fusion Eclipse Mass Spectrometer was used to perform LC-ESI-MS/MS analysis. Briefly, 10 mL of peptide extracts was loaded onto a 2 cm C18 trap column attached to an Ultimate 3000 RSLCnano-HPLC instrument (Thermo, Tewksbury, MA, USA) equipped with an autosampler. Subsequently, peptides were eluted onto a 15 cm analytical C18 column. Samples were loaded at 8 µL/min for 4 min, then a 60 min gradient was applied at 300 nL/min starting from 2 to 35% buffer B (95% acetonitrile, 0.1% formic acid), followed by a 5 min linear gradient to 60%, followed by a 2 min linear gradient to 80%, maintenance at 80% buffer B for 4 min, and a return to 5% over 1 min. Data were acquired using an ion spray voltage of 2.5 kV. MS spectra were acquired using full scans and MS2 scans at 60,000 resolution in the orbitrap with a mass scanning range of 350–2000 *m*/*z*. Maximum injection time was limited to 118 min with a standard AGC target value. A 10 s dynamic exclusion time with a ±10 ppm error tolerance was applied. For specific procedures refer to our previous paper [63].

### 4.8. Proteomic Data Statistics and Analysis

Raw data files acquired from the Orbitrap Fusion Eclipse instrument were loaded onto PEAKS X pro protein database search software (PEAKS studio 10.6 build 20201221, Waterloo, Canada) and protein identification was performed using PEAKS X pro against the UniProt database. Proteins were identified using a mass tolerance of ±10 ppm for intact peptide masses and ±0.02 Da for fragmented ions, with an allowance for three missed cleavages in trypsin digests. Oxidation (M) and deamidated (NQ) were selected as variable modifications, while carbamidomethyl (C) was selected as fixed modifications. Specifically, an automatic decoy database search was performed in PEAKS X, along with a search of the real database. Confident protein identification required at least one unique peptide. The false discovery rate (FDR) of identified proteins was ≤0.01. For protein quantification, a protein was required to contain at least two unique peptides. The quantitative protein ratios were weighted and normalised by the median ratio. Only ratios with *p* <0.05 according to Student’s *t*-test were employed, and only fold changes >2 were considered significant. 

### 4.9. The Kjeldahl Method and BCA Assays

Crude protein content was determined using the Kjeldahl method. Briefly, algal cells were harvested by centrifugation at 3000 rpm for 5 min and washed twice with distilled water. Cells were rapidly dehydrated in an oven at 105 °C for 10 min and dried overnight at 80 °C. Dried cells were sent to Michybio Biotechnology Company to measure crude protein content. To determine the content of dissolved protein, 0.1 g fresh algal cells was resuspended in 1 mL distilled water and sonicated on ice. According to the published method, measurement of soluble protein content was accomplished by a BAC protein detection kit (Michybio, M1806A, Jiangsu, China) [61,64].

### 4.10. Statistical Analysis

For statistical analysis, Student’s *t*-tests were conducted to compare means from replicates, and significance was determined at 0.05, 0.01, and 0.001.

## 5. Conclusions

Due to its simple culture, short growth cycle, and ability to produce economically valuable products, *C. reinhardtii* is considered a green and sustainable cell factory, and an alternative platform for producing recombinant proteins. Although many genetically engineered algal strains expressing recombinant proteins have been reported, inadequate protein yield severely hinders industrialisation. Various measures have been attempted to improve protein production. Our current study employed a novel approach to overcome this barrier. Based on the biological function of the poly(A) tail and poly(A) polymerase, we hypothesised that increased polyadenylate activity would have a positive effect on protein translation and accumulation. After confirming CrePAPS polyadenylation activity, we generated *CrePAPS* OE strains. By characterising the proteome profile, upregulation of ribosomal subunits was observed. Elevated ribosomal assembly implies an overall enhancement in cellular translation. Ultimately, quantification of cell protein content proved that increased polyadenylate activity improves mRNA translation and increases protein accumulation.

## Figures and Tables

**Table 1:** MS/MS profiles and total number of identified unique peptides and proteins.

Strains	No. of MS/MS Fragments	No. of Unique Peptides	No. of Unique Proteins
MT-1	24,711	7131	2262
MT-2	24,745	7185	2187
MT-3	24,760	7013	2188
WT-1	24,687	6814	2452
WT-2	24,711	6931	2376
WT-3	24,732	6982	2399
OE-6-1	24,766	7038	2197
OE-6-2	24,777	7347	2181
OE-6-3	24,762	6818	2203
OE-19-1	24,787	6836	2178
OE-19-2	24,802	6858	2168
OE-19-3	24,849	7513	2199
OE-21-1	24,775	7014	2494
OE-21-2	24,739	6939	2492
OE-21-3	24,790	8293	2553
Total	371,393	15,265	3613

## Supplementary Materials

The following supporting information can be downloaded at https://www.mdpi.com/article/10.3390/md20050276/s1: Figure S1, SDS PAGE detection and silver stain the 5 strains. Lanes 1~5 indecate total protein sample of WT MT OE6 OE19 and OE21. Lanes 6~10 indecate same protein sample as 1~5 which dilute twice the volume. Table S1, Primers used in this study. Table S1, Primers used in this study; Table S2, Quantitative amount of identified protein; Table S3, Screening of differentially accumulated proteins (DAPs); Table S4, Ratio content of simultaneously detected proteins; Table S5, Screening of proportionally altered proteins (PAPs); Table S6, GO enrichment results for biological process, molecular function, and cellular component categories of up-DAPs; Table S7, GO enrichment results for biological process, molecular function, cellular component categories of up-PAPs; Table S8, KEGG enrichment results for up-DAPs and up-PAPs.
