# Supplementary material for: Overexpressing CrePAPS Polyadenylate Activity Enhances Protein Translation and Accumulation in Chlamydomonas reinhardtii
Source: Mar Drugs. 2022 Apr 21;20(5):276. doi: 10.3390/md20050276 (PMC9147819; doi:10.3390/md20050276)
Supplement: Supplementary file 1 [file marinedrugs-20-00276-s001.zip › manuscript-03/table S1.pdf]

Table S1. Primers used in this study.

Primer ID	Primer Sequence
ASZU15	CCCATATGATGGCAGTTGCAGACCCAAAC
ASZU16	GGGGTACCTCAAGAGCCAGCCGTAGGCG
ASZU17	GCCGACAAGGACCCCGACGCA
ASZU18	CATCATCCTGGTTTGGCTGC
SP8	ATTCAGGTGAACACTTGGGC
SP9	CTTCATGTGTGGTCGTTTGG
ASZU40	AGACACTTATTTACACCTGGACATG
ASZU41	CAGACGGCTGATGGAATTGA
ASZU42	GGCGTCCCAGGCGGATGAGC
ASZU43	AACTGCTCTCCTATCACCATACCAC
ASZU44	GAGAGCAGTATCTTCCATCCACCGC
ASZU45	CAAGGACAAGGTGCCCAACG
ASZU46	CAACTCGCTGCTGCTCCACA
ASZU47	ACGTCTACACCTTCGGCTCGTAC
ASZU48	GGCGTACAGGATGTCGATTTG
ASZU50	ATGGCAGTTGCAGACCCAAAC
ASZU51	TCAAGAGCCAGCCGTAGGCGAA

Note: ASZU15&ASZU16 amplicon is used to construct *pJ1DCF-CrePAPS* vector; ASZU17&ASZU18 is used to detect positive transformants; SP8&SP9 is used to identify mutant; ASZU40&ASZU41 is used for determining *CrePP2A* gene expression; ASZU42&ASZU43 is used for determining internal *CrePAPS* gene expression; ASZU42&ASZU44 is used for determining external *CrePAPS* gene expression. ASZU45&ASZU46 is used for *CrePP2A* Semi-quantitative detection; ASZU47&ASZU48 is used for *CrePAPS* Semi-quantitative detection; ASZU47&ASZU48 amplicon is used to construct vector *pEASY-E1-CrePAPS*.
