# Supplementary material for: Overexpressing CrePAPS Polyadenylate Activity Enhances Protein Translation and Accumulation in Chlamydomonas reinhardtii
Source: Mar Drugs. 2022 Apr 21;20(5):276. doi: 10.3390/md20050276 (PMC9147819; doi:10.3390/md20050276)
Supplement: Supplementary file 1 [file marinedrugs-20-00276-s001.zip › marinedrugs-165793 figure S1.pdf]

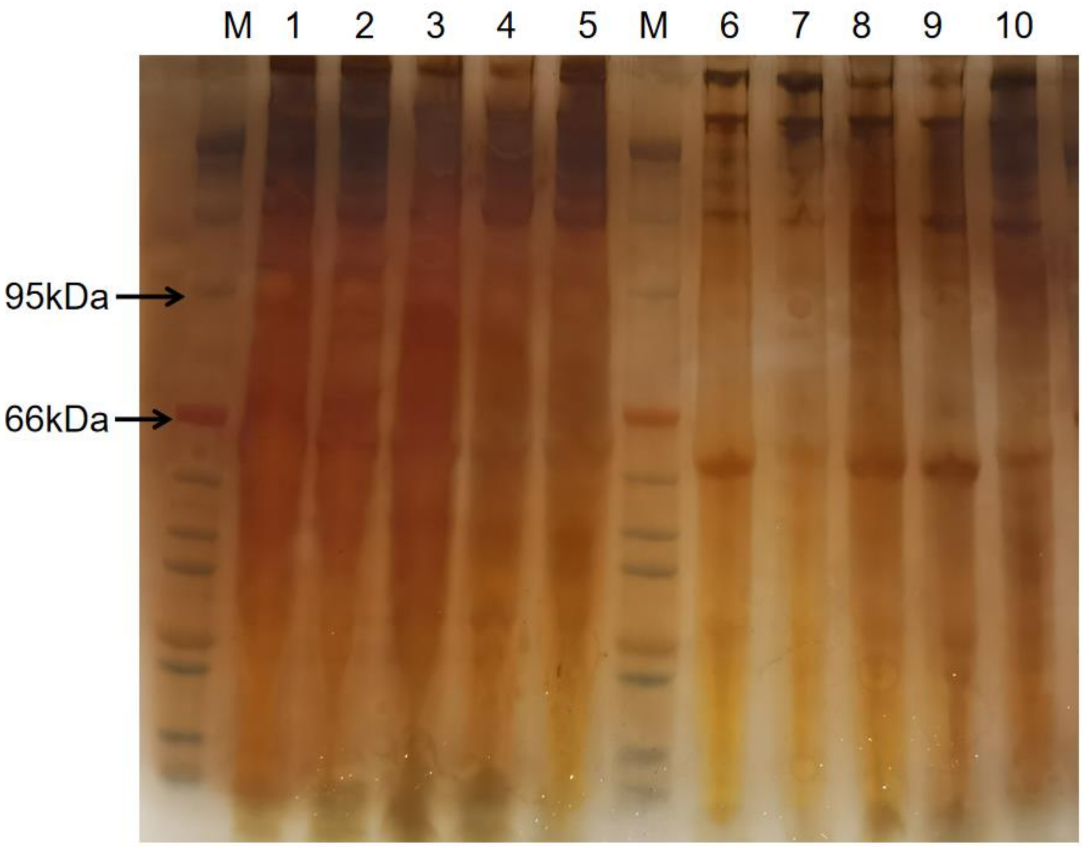

Figure S1. SDS-PAGE detection and silver-stain the 5 strains. Lanes 1~ 5 indecate total protein sample of WT，MT，OE6，OE19 and OE21. Lanes 6~ 10 indecate same protein sample as 1~ 5 which dilute twice the volume.
